# Supplementary material for: Electrocatalytic Reduction of CO2 in Water by a Palladium-Containing Metallopolymer
Source: Nanomaterials (Basel). 2022 Apr 2;12(7):1193. doi: 10.3390/nano12071193 (PMC9000595; doi:10.3390/nano12071193)
Supplement: Supplementary file 1 [file nanomaterials-12-01193-s001.zip › nanomaterials-1650625-supplementary.pdf]

SUPPLEMENTARY MATERIALS

Electrocatalytic reduction of CO_2 in water by a palladium-containing metallopolymer

Marcos F. S. Teixeira[1*], André Olean-Oliveira[1], Fernanda C. Anastácio[1], Diego N. David-Parra[1], Celso X. Cardoso[2]

Keywords: metallopolymer; electroreduction of dioxide carbon; electrocatalysis

1. M. F. S. Teixeira (🖂); A. Olean-Oliveira; F. C. Anastácio; D. N. David-Parra
Department of Chemistry and Biochemistry - School of Science and Technology
Sao Paulo State University (UNESP)
Rua Roberto Simonsen, 305
CEP 19060-900 - Presidente Prudente, SP, Brazil.
Tel.: +55 18 3229 57 49; Fax: + 55 18 3221 56 82:
E-mail address: marcos.fs.teixeira@unesp.br (Teixeira, M. F. S.)
orcid.org/0000-0001-9355-2143
2. C. X. Cardoso
Department of Physics - School of Science and Technology
Sao Paulo State University (UNESP)

Figure S1

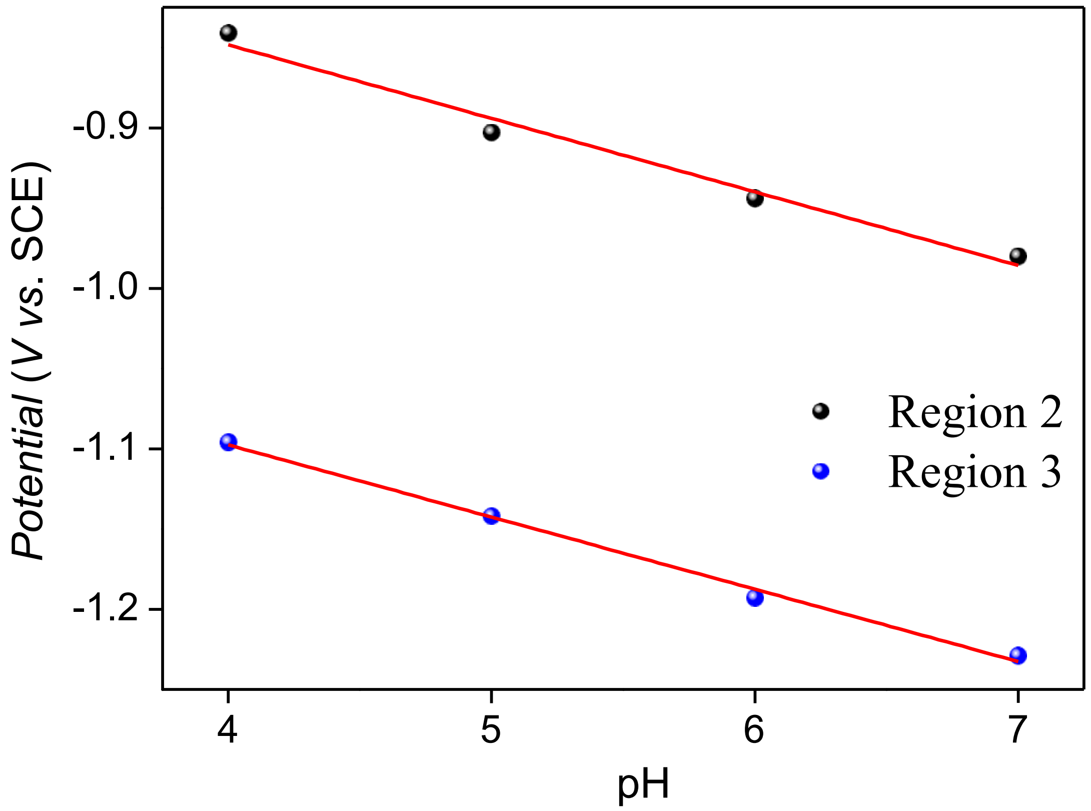

Figure S1. Potential versus pH curves in 0.50 mol L^{-1} KCl solution saturated with CO_2 at a scan rate of 25 mV s^{-1}.
